# MultiSenseBadminton: Wearable Sensor–Based Biomechanical Dataset for Evaluation of Badminton Performance

**DOI:** 10.1038/s41597-024-03144-z

**Published:** 2024-04-05

**Authors:** Minwoo Seong, Gwangbin Kim, Dohyeon Yeo, Yumin Kang, Heesan Yang, Joseph DelPreto, Wojciech Matusik, Daniela Rus, SeungJun Kim

**Affiliations:** 1 Gwangju Institute of Science and Technology, School of Integrated Technology, Gwangju, 61005 South Korea; 2 Massachusetts Institute of Technology, CSAIL, Cambridge, MA 02139 USA

**Keywords:** Information technology, Software

## Abstract

The sports industry is witnessing an increasing trend of utilizing multiple synchronized sensors for player data collection, enabling personalized training systems with multi-perspective real-time feedback. Badminton could benefit from these various sensors, but there is a scarcity of comprehensive badminton action datasets for analysis and training feedback. Addressing this gap, this paper introduces a multi-sensor badminton dataset for forehand clear and backhand drive strokes, based on interviews with coaches for optimal usability. The dataset covers various skill levels, including beginners, intermediates, and experts, providing resources for understanding biomechanics across skill levels. It encompasses 7,763 badminton swing data from 25 players, featuring sensor data on eye tracking, body tracking, muscle signals, and foot pressure. The dataset also includes video recordings, detailed annotations on stroke type, skill level, sound, ball landing, and hitting location, as well as survey and interview data. We validated our dataset by applying a proof-of-concept machine learning model to all annotation data, demonstrating its comprehensive applicability in advanced badminton training and research.

## Background & Summary

Recent advances in wearable sensors have enabled the accurate recognition of human actions at a highly detailed level^1,2^. This, in conjunction with modern AI techniques, has facilitated the analysis of high-level actions in various fields, such as fall detection^3–7^, sports training^8–15^, healthcare^16–19^, assistive technologies for people with disabilities^20,21^, and rehabilitation^22,23^. While other fields focus on reducing the number and size of sensors used in research, the sports industry is adopting multimodal sensors to gain a comprehensive understanding of player movements and physical states. These sensors facilitate various analyses, from simple posture classification to performance analysis, and include inertial measurement units (IMUs)^24–29^, eye trackers^30–32^, pressure sensors^33,34^, skeleton tracking sensors^35–40^, electromyography (EMG) sensors^41,42^, and capacitive sensors^43^.

Multimodal sensors provide quantitative and objective performance data on players’ performance, a feature that can be exploited to maximize the training effect for each individual. By providing in-depth analyses into joint movement patterns^15,35,36,38,44^, muscle activation^14,41^, and gaze movements^45^, sensor-based tools offer a level of feedback that traditional coaching methods struggle to achieve^46^. The role of the human coach, which traditionally involves meticulous tracking of a trainee’s every motion, can be partly automated through these applications^47–49^. This shift allows coaches to focus on providing personalized feedback, including tracking a player’s progress, identifying areas for improvement, strengthening specific weaknesses^14^, or even injury prevention^50–52^. These AI-assisted coaching methods offer a more accessible and inclusive approach to training, granting individuals 24/7 access to expert feedback^49^, or supplying an AI coaching service for those who might otherwise not have access to a coach^36,38,40,45,47,53–56^. Such ubiquity in training access ensures that a larger number of individuals, irrespective of geographical constraints or other limitations, can benefit from expert guidance and training programs.

Likewise, several AI- and sensor-based diagnostic systems have been proposed for badminton training^8–14^. Since badminton performance largely depends on the correct execution of each stroke, which requires quick and complex reflexes, sensor-based action analysis can be especially advantageous. Specifically, performing an effective badminton stroke requires proper stance, power control, and arm speed^12^, all of which are difficult for a human coach to monitor simultaneously. In particular, for beginners who are not yet familiar with basic badminton movements, acquiring the proper swing posture and power control can often require a considerable period of training, sometimes extending over several months. Therefore, by utilizing wearable sensors and AI technology to collect data from players of various skill levels, a system could be developed that not only assists in the training process but also provides an objective metric to complement a coach’s assessment.

Despite the benefits and prevalence of computer-assisted applications in badminton training, there is a limited amount of publicly accessible badminton action data available for training system development. Badminton datasets typically fall into two categories: individual stroke data collection in controlled settings, and strategy analysis based on real-world match videos (see Table 1). However, most publicly available datasets^57–60^ focus on match data between professional players, with an emphasis on tactical aspects. These aspects include predicting an opponent’s shuttlecock trajectory and stroke types^59,60^, as well as detecting strokes and identifying players’ bounding boxes^57,58^. The unpredictable trajectory, speed, and timing of the shuttlecock in real-match scenarios, coupled with the players’ dynamic movements, make modeling and evaluating individual strokes particularly challenging, as it requires accounting for previous strokes and the opponent’s actions.

**Table 1:** Comparison of the MultiSenseBadminton dataset[64] with existing public and non-public badminton datasets: In the “Context” column, “C” denotes collecting badminton data in a constrained, controlled environment, while “F” indicates data collection in the field during actual competitive play between two or more players.

Datasets	Years	Modalities	Target Population	#Subject	#Videos	Tasks	Context	Publicly Available
Badminton IMU^61^	2016	IMU	PR.	12	—	CLA.	C	No
Badminton Olympic^57^	2018	Videos	PR.	—	10	LOC.&CLA.	F	Yes
BadmintonPose^11^	2019	Videos	PR.	1	12	CLA.	C	No
Badminton Plantar^9^	2019	Foot pressure, Skeleton	PR.&AM.	20	—	STAT.	C	No
BAR^62^	2020	IMU	PR.&AM.	11	—	CLA.&ASS.	C&F	Yes
Badminton EMG^14^	2021	Videos, EMG (arm)	PR.	4	—	ASS.	C	No
BadmintonDB^58^	2022	Videos	PR.	2	9	CLA.	F	Yes
BadmintonACC^13^	2022	Accelerometer, Gyroscope	AM.	5	—	CLA.	C	No
Badminton Landing Task^63^	2022	EMG (leg), Skeleton	AM.	34	—	STAT.	C	No
ShuttleSet^59^	2023	Videos	PR.	27	44	CLA.	F	Yes
ShuttleSet22^60^	2023	Videos	PR.	35	58	CLA.	F	Yes
MultiSenseBadminton (Ours)^75^	2024	Videos, Skeleton, Eye gaze, Foot pressure, EMG (arm, leg)	PR.&AM.	25	225	CLA.&ASS.	C	Yes

In the “Task” column, “CLA.”, “ASS.”, “STAT.” stands for action classification, action quality assessment, and cross-player statistical analysis. In the “Target Population” column, “PR.” refers to professional players, and “AM.” refers to amateur players.

In contrast, individual stroke data collection in controlled setting, which is our primary area of focus, provides opportunities for stroke classification^11,13,61,62^, statistical comparisons across varying expertise levels^9,63^, and in-depth evaluation of each stroke^14,62^. Given the context in which the individual stroke data is used, gathering it in a stable environment is crucial, allowing to focus entirely on the mechanics of badminton movements and accurately capture the full dynamics. Specifically, the controlled environment for stroke data collection increases the potential to gather diverse biometric and motion data through wearable sensors. This includes IMU sensor data^12,13,61,62,64^, motion capture data^9,63^ as well as collecting biometric information like electromyography^14,63,65^ and foot pressure^9^. Such an environment also supports the use of cameras for analysis^10,11^.

However, there is a gap in the available datasets, particularly in terms of assessing the quality of badminton strokes. Existing research has not fully addressed key elements of badminton swings, such as the players’ skill levels, the final position of the shuttlecock after the swing, the quality of impact during the swing, and the hitting point. Furthermore, although most datasets focus mainly on data from one or two types of sensors, fully understanding player performance variations requires combining data from multiple sources, including motion, foot pressure, and muscle activity, to get a comprehensive view of stroke quality.

To address the gap identified in previous research, our study concentrated on evaluating the quality of individual strokes for players at various skill levels rather than focusing on tactical strategies. This approach involved collecting data in controlled environments, where players executed strokes using a shuttlecock launcher^63^ while being monitored with various sensors. Our research specifically concentrated on collecting data for two primary strokes taught in beginner badminton club courses: the forehand clear and the backhand drive^14,66–68^. We aimed to assess how the quality of each player’s stroke varies with different postures. For this, we collected over 150 swing data points per stroke type, involving players of various skill levels^9^. Therefore, we collected a multimodal badminton swing dataset incorporating both motion and physiological data, including full-body motion, foot pressure, gaze, and muscle activation data.

Our dataset surpasses previous approaches by incorporating a diverse range of data sources not included in existing badminton datasets. Our data collection setup, including the environment and sensor configuration, was developed based on insights from interviews with badminton experts. Our dataset encompasses five types of sensor data streams captured simultaneously, along with expert interviews, surveys, and annotated data. The dataset also includes video recordings from different point of view (Front, Side, Whole, Eye, and Eye with Gaze Overlay). The annotation data includes stroke type, skill level, ball landing location, shuttlecock sound, and hitting position. By compiling this comprehensive dataset, we provide a detailed representation of badminton strokes and related characteristics. This dataset can be leveraged to develop training programs, performance analysis techniques, and coaching strategies in the sport of badminton.

Our research additionally introduces an initial framework for utilizing machine learning with our dataset in the Technical Validation section. This section outlines a methodology that includes preprocessing and feature extraction, emphasizing the suitability of our dataset for machine learning applications. This includes providing examples of classifying stroke type, skill level, horizontal and vertical landing position, hitting point, and stroke sound. We reported the accuracy of our annotations with state-of-the-art machine-learning techniques. To facilitate usage by a wide audience, including those not specialized in deep learning, we have provided examples and made our deep learning pipeline source code openly accessible on the project’s GitHub page.

## Methods

### Dataset design

To build a badminton action dataset designed specifically to address the needs of the badminton coaching field, we engaged three professional badminton coaches from a local club that boasts a membership of over 50 individuals. Each coach had undergone professional training and had a minimum of five years of coaching experience (Female: 1; Age: Mean = 36.7, SD = 11.3; Years of Experience: Mean = 11, SD = 5.1). Our main aim was to extract their knowledge, focusing particularly on insights related to the overall training process. This knowledge would then guide us in selecting an appropriate sensor set and designing a dataset to facilitate player performance analysis and feedback.

We centered our discussions around critical elements that expert coaches pay attention to when teaching swing techniques. This included their standardized training processes, strategies for providing feedback, and the strategies used for executing stroke actions. To prepare for the interviews, we sent the questions to the coaches in advance. Each interview lasted roughly an hour, and each coach received a compensation of $80 for their participation.

The following subsections provide an aggregated summary of the interview responses. While our dataset entails the complete answers to a total of six questions, this paper specifically highlights the four questions that directly influenced the design of the dataset (see Table 2). These four questions primarily focus on the types of data and annotations that should be included for the analysis of badminton stroke actions. The full set of six questions, providing comprehensive insights into the coaches’ views on the applicability of AI-based coaching systems, challenges faced in current training methodologies, and thoughts on coaching within a virtual environment, can serve as a useful guide for future dataset design efforts.

**Table 2:** Summary of Interview with Badminton Coaches.

Questionnaire List	Summary of Responses
1. What is the most important skill to teach during badminton training?	Grip, Stance, Swing, Step
2. What is the criterion for evaluating the success of badminton training?	Location of the hitting point,
	Landing point of the ball (trajectory),
	Stroke accuracy (sound),
	Ball speed
3. How do you give feedback to trainees during training?	Verbal comments, Demonstration,
	Self-video recording,
	Comparison using videos of skilled players
4. What is the important data for making an effective badminton shot?	Appropriate timing of hits, Grip force,
	Gaze, Body tracking, Hand pressure,
	Foot pressure, Muscle activation,
	Racket tracking, Ball tracking

#### Question 1. What is the most important skill to teach during badminton training?

##### Summary of responses to Question 1

The coach highlights the significance of grip, posture, swing, and step in badminton training. Grip training is emphasized as a continuous process to enhance racket control and precision. For beginners, the coach prioritizes teaching proper posture, followed by improving swing accuracy and shot execution. Maintaining the correct swing involves generating power from the rotation of the torso, along with the coordinated movement of the arm and wrist. The evaluation of shot accuracy focuses on hitting the intended target or clearing the net correctly. In terms of step, the coach emphasizes positioning the dominant foot underneath the shuttlecock’s expected landing spot and highlights the importance of the split step for quick post-shot preparation. Overall, the coach underscores the importance of these elements in effective badminton training.

#### Question 2. What is the criterion for evaluating the success of badminton training?

##### Summary of responses to Question 2

According to the interviews, coaches evaluated the badminton training process based on several key factors. First, the point of impact at which the ball hits the racket is evaluated-specifically, whether the ball is in front of the body when hitting. Second, the trajectory and landing location of the ball are assessed to ensure that it travels through the target distance and direction. Third, the accuracy of the stroke, which is determined by assessing whether the ball makes contact with the center of the racket and the sound produced during this interaction, is evaluated. Finally, the speed of the ball is also monitored.

#### Question 3. How do you give feedback to trainees during training?

##### Summary of responses to Question 3

Coaches typically employ four main methods to provide feedback to badminton students. First, the coaches provide verbal feedback to the students to inform them of their performance and whether they have executed the correct stroke. If a student still has difficulty maintaining the proper posture or executing the correct movement, the coach may demonstrate an example to clarify the appropriate form. Additionally, some coaches may use video feedback to help students track their progress or observe the correct motion of a skilled player. However, while video feedback can be effective for some students, it may not always be the most useful tool for everyone. Although videos can provide a visual aid for learning, many students may find it difficult to fully grasp the technique without the opportunity to physically practice and experience the movements themselves. In particular, providing feedback on concepts that are difficult to understand visually, such as the application of force or shifting one’s center of gravity, can present challenges for coaches. Therefore, coaches may need to tailor their feedback strategies to suit individual learning styles and preferences to optimize student learning and development during badminton training.

#### Question 4. What are the important data for an effective badminton stroke?

##### Summary of responses to Question 4

Several factors contribute to the effectiveness of a badminton shot, including swing accuracy, footwork, and gaze processing, all of which collectively help to ensure the shuttlecock is hit at the optimal timing and trajectory. Executing a fast and precise swing entails a sequence of actions: visually tracking the ball, extending the arms, stepping towards the target, and delivering a forceful impact. Holding the racket correctly is also crucial in badminton to generate impact during a shot, enabling greater wrist flexibility and range of motion to execute various types of shots. Proper footwork is essential for maintaining appropriate body positioning and stable shots, relying on precise balance control and efficient movement patterns, which should be attentively practiced to direct the shuttlecock towards the intended direction at the desired speed. Consequently, sensor-based analysis of badminton strokes requires the collection of data on various factors, including tracking racket position, monitoring ball trajectory, measuring hand pressure, tracking eye movements, monitoring body and foot positioning, assessing foot pressure, and analyzing muscle activity.

#### Interview-based dataset design

In our study, we established the sensor set, data collection environment, target strokes, and annotation data through interviews with experts. The selection of the most suitable sensors, guided by insights from Question 4 in the interviews, emphasized the need for sensors that capture crucial data without impeding natural badminton swing movements. Therefore, we opted for a non-invasive, comprehensive sensor set suitable for players of various skill levels, including eye gaze tracking, EMG, IMU-based body tracking, and foot pressure sensors.

To avoid altering the racket’s weight or feel with attached sensors, we chose motion tracking technology worn on the hand (Perception Neuron studio) to measure racket movements. This decision was informed by studies in racket sports^69–73^, where IMU sensors on the hand provided stroke classification performance comparable to sensors on the racket^74^, and in some cases, even superior correlation with player performance-related measures^72^. This approach allows us to gather essential swing information via IMU sensor-based data from the hand, maintaining the racket’s natural feel and balance during play and offering proxy measures for racket dynamics.

For the data collection setting, we drew inspiration from typical badminton training environment where a coach throws shuttlecocks for the trainee to return, often providing real-time posture correction and feedback. To replicate this training environment consistently in our study, we employed a shuttlecock launcher for collecting badminton stroke data^63^. We calibrated the launcher to consistently release shuttlecocks at the same angle for each stroke type, which enabled the collection of comparable swing data across different participants. This setup was instrumental in gathering data on how players of various skill levels respond to the same shuttlecock trajectory, thereby facilitating an analysis of the diversity in players’ responses to uniform strokes. To thoroughly observe the participants’ posture and the shuttlecock’s trajectory, we installed three external cameras, each capturing a unique view. In addition, an eye tracker camera was utilized to record sound.

For our target strokes, we concentrated on two basic strokes essential for beginners in badminton training: the forehand clear and backhand drive. This focus was driven by the goal of our dataset, which is to evaluate the quality of individual strokes for building a badminton training system. By narrowing down to these fundamental strokes, we aimed to collect over 150 data points per participant, focusing on how players of different skill levels react to shuttlecocks with the same trajectory and how their responses vary. Drawing on answers from Question 2 of our expert interviews, we established criteria to evaluate each badminton stroke and annotated these criteria to build a dataset on stroke quality. Our annotations included aspects such as the skill level of each player, the horizontal and vertical location of each strokes, the hitting point, and the sound quality produced during the hit. By concentrating on these specific strokes and detailed annotations, we sought to provide a comprehensive dataset that would offer insights into the nuances of stroke execution and quality across various skill levels in badminton.

### Ethics statement for the multisensebadminton dataset

The development of the MultiSenseBadminton dataset received ethical clearance from the Institutional Review Board (IRB) at the Gwangju Institute of Science and Technology^75^. This project was approved under the protocol code 20220628-HR-67-20-04 on July 21, 2022.

Upon arrival at the data collection site, participants were presented with consent forms. These forms required thorough reading and written agreement from participants, confirming their willingness to contribute to the data collection process. A critical aspect of the MultiSenseBadminton dataset is its public availability. As such, explicit consent was also obtained for the public release of data that includes personally identifiable information (PII), specifically the video recordings of participants performing badminton swing. The videos have undergone mosaic or blur processing for participant confidentiality. The final MultiSenseBadminton dataset comprises video, annotation, personal information and sensor data from all 25 participants who participated in the study, each of whom consented to the release of their personal data for public access.

### Participants

In this study, data were collected from 25 participants (20 males and 5 females) aged between 18 and 52 years (*Mean* = 26.8 years, *SD* = 6.59 years). The basic physical conditions of the participants were as follows: weight 48–108 kg (*Mean* = 76.4 kg, *SD* = 14.6 kg); height 160–190 cm (*Mean* = 174 cm, *SD* = 8.33 cm) (Shown in Table 3). The participants’ training experience varied between 0 and 22 years (*Mean* = 3.96 years, *SD* = 6.9 years). All participants demonstrated dominance in one hand and confirmed that they predominantly use this hand in badminton. Before data collection, the participants were briefed about the use of multiple wearable sensors and agreed to participate in the study. After collecting data, the participants were paid $40 for participating. All participants consented to data disclosure, and data for all subjects were included in our dataset.

**Table 3:** Demographics and Training Experiences of Subjects: The self-reported skill level was recorded on a 7 Likert scale; the higher the Likert value, the higher the skill level.

ID	Age [years]	Gender [M|F]	Weight [kg]	Height [cm]	Dominant hand	Dominant leg	Training time [years]	Self-reported skill level [7 Likert scale]
1	18	F	58	165	R	R	0	1
2	24	F	65	164	R	R	0	1
3	25	M	90	181	R	R	0	2
4	21	M	65	175	R	R	0	1
5	21	M	103	166	R	R	0	2
6	19	M	90	187	R	R	0	2
7	23	M	61	164	R	R	0	2
8	24	M	85	170	R	R	0	1
9	28	M	90	190	R	R	0.83	3
10	26	M	64	171	R	R	1	5
11	24	M	63	174	R	R	0	1
12	52	F	58	160	R	R	14	7
13	26	F	68	166	R	R	2.5	5
14	26	M	70	169	L	L	0.5	4
15	28	M	72	186	R	R	18	7
16	38	M	83	170	R	R	2	4
17	28	M	77	180	R	R	0.25	2
18	24	F	48	161	R	R	0	2
19	31	M	80	182	R	R	2	3
20	28	M	82	180	R	R	19	7
21	25	M	108	166	R	R	14	7
22	24	M	75	178	R	R	1	3
23	29	M	73	179	R	R	2	4
24	32	M	90	176	R	R	0	2
25	28	M	92	180	R	R	22	7

### Sensors and data collection framework

The sensor collection framework used in our study is an adaptation of the ActionSense framework^76^. The original framework encompassing codes, a graphical user interface (GUI), and sensor visualization capabilities was tailored for human-activity data collection from wearable devices during kitchen activities. We therefore modified this framework to align with our unique sensor set and dataset design. This involved customizing the GUI and real-time data visualization features of ActionSense for *in-situ* monitoring and time-synchronized annotation during data collection.

**Fig. 1:**
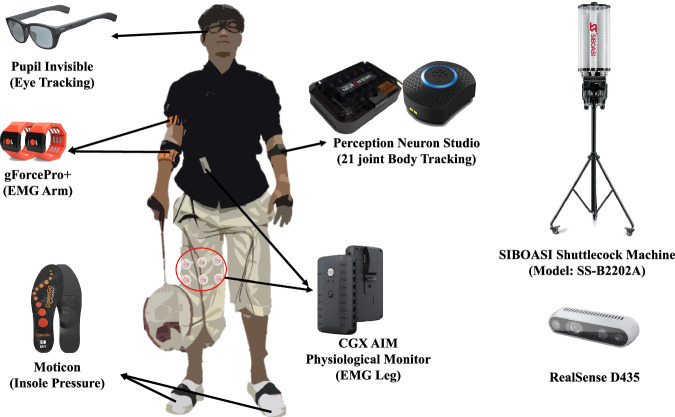
The sensors used for data collection during the experiment.

Our study utilized five types of wearable sensors: eye tracking, body tracking, foot pressure, and EMG sensors. We supplemented these with three cameras and a shuttlecock launcher for comprehensive data collection focused on badminton stroke analysis (see Fig. 1). Each sensor’s data stream was integrated into the overarching ActionSense framework by connecting their respective Python API or stream layer API, thereby facilitating data import via TCP/IP communication.

To ensure easy data manipulation and seamless integration, we opted to save the collected data in an HDF5 file format^77^. This format offers cross-platform and cross-language compatibility, rendering it a versatile choice for storing and accessing large volumes of scientific data. Furthermore, the HDF5 format allows for the hierarchical organization of multimodal heterogeneous data such as sensor readings. Given its capability for processing large data in concurrent threads and parallel I/O, the HDF5 format is particularly suitable for our data-rich configurations that involve five wearable sensors and three cameras in the simultaneous data stream channels.

**Fig. 2:**
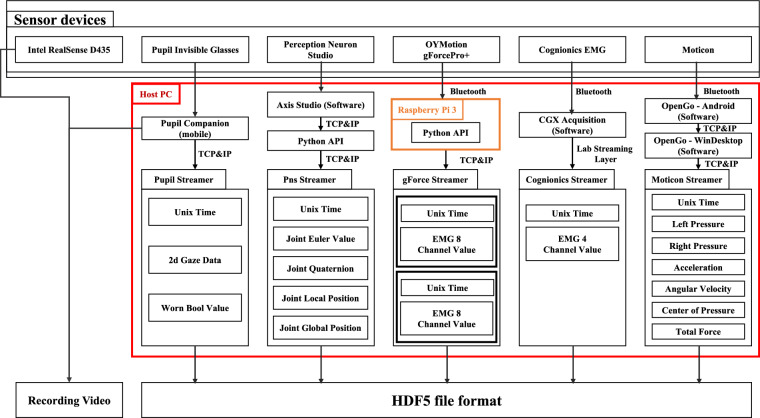
Sensor Data Collection Framework.

We collected all data using Unix time to assist in analyzing the temporal relationship between different sensor data points. The structure of the sensor acquisition framework is illustrated in Fig. 2. Notably, even in the event of a sensor disconnection during data collection, the entire framework continues data acquisition seamlessly.

#### Eye tracking (Pupil Invisible Glasses)

In our study, we utilized Pupil Invisible glasses to collect 1) information on whether gaze data were successfully received; 2) 2D gaze data (horizontal and vertical); 3) ambient sound that participants were subject to; and 4) first-person videos through eye camera. The Pupil Invisible glasses are a wearable eye-tracking system designed to resemble a regular pair of glasses. The system includes two inner cameras on the frame to track eye movements, an exterior camera on the left temple with a wide field of view to record the environment, and a USB-C connector on the right temple that connects to a smartphone running the tracker application. The eye-tracker output comprises recorded videos displaying the participant’s gaze position and coordinates relative to the outside image. While the Pupil Invisible glasses autonomously estimate gaze data via an embedded convolutional neural network algorithm^78^, eliminating the need for traditional calibration, we adopted an additional step to enhance data robustness. Before collecting swing data, participants were asked to focus on monitor corners to verify the accuracy of the machine-predicted gaze data. The system weighs only 46.9 g and comes with a lens kit; it can therefore be fitted with separate −3 to +3 diopter lenses in steps of 0.5. Network streaming was performed using a OnePlus 8 smartphone, and data were collected via a wired connection between the phone and the glasses. This system provides robust gaze estimation in any environment, including outdoor settings, which is essential for this study. We collected data on Unix time, 2D gaze, and gaze worn value in real time at a sampling rate of 30 Hz using Pupil Invisible Companion and saved these data in HDF5 format.

#### Body tracking (perception neuron studio)

For the measurement of joint angles and positions in our study, we employed the Perception Neuron Studio application. Owing to its data stream reliability as reported in motion capture studies^79–83^, this system is frequently utilized for collection of human motion data. Furthermore, it has applications in motion balance and ergonomic analyses^84,85^. The system estimates body joint positions and angles based on the IMU. It is comprised of 17 trackers, each measuring 12.5 mm Ã— 13.1 mm Ã— 4.3 mm and containing a triaxle gyroscope (2000 DPS), magnetometer, and accelerometer (32 g). All 17 trackers were affixed to the body using a belt positioned at the specific joints designated for each sensor. Perception Neuron Studio also offers sensor calibration through its third-party software, requiring three poses for calibration: the T-pose, squat, and N-pose.

The data stream, including the local Euler angle, local quaternion, and local position, can be transmitted in real time via network communication. However, it is worth noting that owing to inherent limitations in the IMU, the global position may drift over time. In our study, we collected real-time data on the local position, local Euler angle, and local quaternion at a sampling rate of 96 Hz using Perception Neuron Studio’s Python API. We used these data to calculate the participant’s global position. As shown in Fig. 2, the resulting HDF5 file contains these four types of data.

#### Muscle activity (gForcePro+, Cognionics AIM)

We collected muscle activity data from both the dominant arm and dominant leg. As all participants held the racket with their dominant arm, the collected data allows us to scrutinize the muscle activity and force application timing of the racket-holding hand. We also included muscle activity data for the dominant leg based on a prior study indicating greater muscle activation in the dominant leg^86^.

To monitor electromyography on the upper and lower parts of the dominant arm, we used the gForcePro + armband. This device communicates using the Bluetooth Low Energy (BLE) 4.2 Standard with a range of up to 10 meters, while being capable of measuring muscle activity across eight channels at a maximum sampling rate of 1000 Hz. We captured data from the following muscles: biceps brachii, triceps brachii, brachioradialis, flexor carpi ulnaris, and flexor digitorum superficialis. Access to read the raw EMG data of the gForcePro + API was provided through a Raspberry Pi 3, which was subsequently integrated into the main computer’s Python framework using TCP/IP communication as the API supports Linux-based environment only. Given that Unix time was retrieved alongside the data, no further adjustments for data latency were necessary during the integration process.

**Fig. 3:**
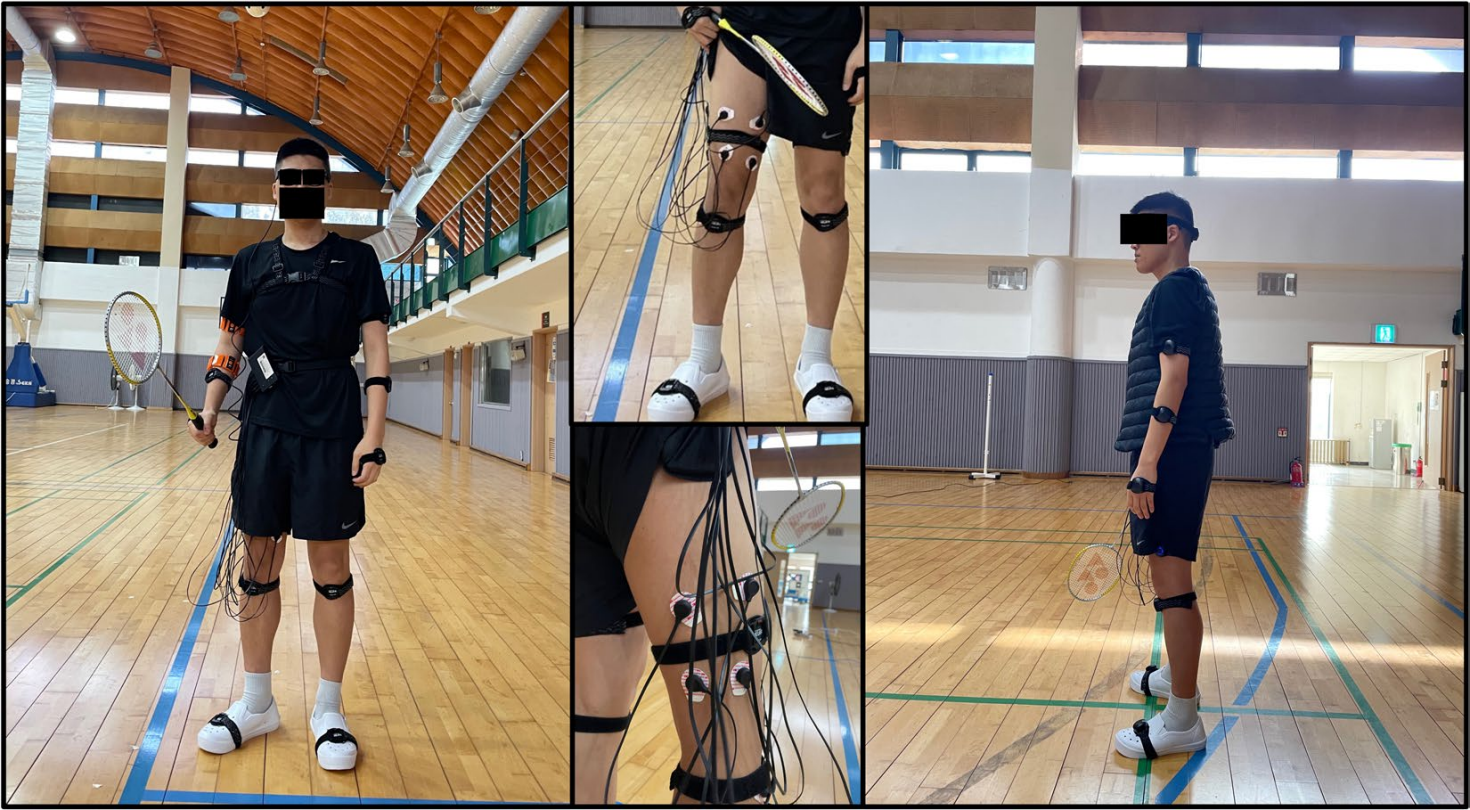
The participant’s appearance while wearing the sensors. Left: front view; middle: position of electrodes attached to the dominant leg; right: side view (The individual depicted in this figure has provided informed consent for the open publication of their image.).

To measure muscle activity in the dominant leg, we utilized AIM, a physiological monitoring device from Cognionics. AIM measures muscle activation using EMG and collects wireless data through the CGX acquisition software. These data were then integrated into Python via the lab streaming layer. Previous research reported that the rectus femoris and vastus medialis muscles exhibit the highest EMG and integrated EMG (IEMG) activity during badminton strokes^87^. Based on these findings, we attached the SkinTact electrodes to the participant’s dominant leg (see Fig. 3 (middle) for detailed contact positions), and CGX software was used to read EMG data at a sampling rate of 500 Hz (channel 1 for rectus femoris; channel 2 for vastus medialis; channel 3 for vastus lateralis; and channel 4 for biceps femoris).

#### Foot-pressure sensor (Moticon)

We adopted Moticon insole sensors to measure foot pressure during badminton strokes. These sensors were wireless and equipped with 16 plantar pressure sensors and a 6-axis IMU, which captured both static and dynamic plantar pressure data as well as 3-axis acceleration and angular rate data. Real-time transmission of data was possible with a maximum sampling rate of 100 Hz through wireless connection to a computer or mobile device, and onboard recording was also an option for offline analysis. To ensure accurate measurement, the OpenGo android application was employed for calibration prior to data collection. The four-step calibration process included slow walking, standing still, rocking the center of gravity of the body back and forth, and rocking the center of gravity of the body left and right.

#### Shuttlecock launcher (SIBOASI SS-B2202A)

**Fig. 4:**
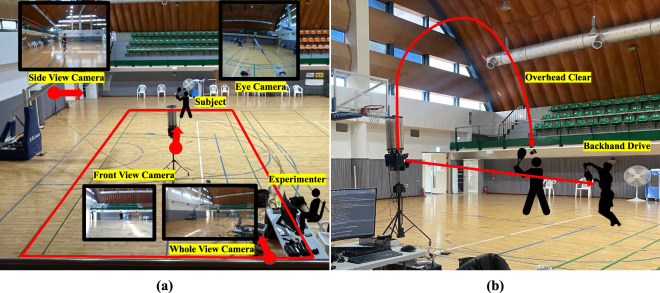
Data Collection Environment. Left: Positions of the camera, the subject, and the analyst; Right: type of task.

In this study, a shuttlecock launcher manufactured by SIBOASI was used to launch shuttlecocks following the same trajectory, ensuring consistency across trials shown in Fig. 4b. The launcher has a weight of 40 kg and can store up to 180 shuttlecocks. The device allows the user to adjust the frequency of the ball launches, with options ranging from 1.2 to 4.5 seconds. The speed of the ball can also be adjusted within a range of 20 to 140 km/h. Additionally, the launcher has a maximum elevation angle of 38 degrees and a horizontal angle of 30 degrees. The device can be controlled remotely using a mobile application or remote control and can launch a variety of ball types, including net balls, smash balls, flat drives, horizontal serves, and vertical serves. The launcher features several modes, such as fixed-point ball, random ball, and combination ball modes, which enable the user to direct the ball to specific locations or deploy randomly. It is also possible to alternate between two ball types for continuous practice. In the fixed-point ball mode, we were able to select a specific location for the shuttlecock to be directed, within the horizontal 0–60 and vertical 10–60 range. For the purpose of our data collection, we set the launcher to horizontal 30 and vertical 50 for forehand clear. We set the launcher to horizontal 15 and vertical 30 for right-handers performing the backhand drive. For left-handers, we adjusted the settings to horizontal 45 and vertical 30. This allowed us to target specific areas on the court.

#### Cameras

As mentioned earlier in the Dataset Design section, a total of three cameras were used to record movements from the front, side, and whole views to obtain information on the participant’s stroke. The placement of the camera and the positions of the participants are shown in Fig. 4a. The front and side cameras recorded at a resolution of 640 × 480 pixels and a frame rate of 30 fps. The whole-view camera recorded at a higher resolution of 1920 × 1080 pixels, with the same frame rate. Furthermore, an eye camera mounted on Pupil Invisible glasses recorded the participant’s first-person perspective at a resolution of 1080 × 1088 pixels and a frame rate of 30 fps, and simultaneously recorded sound. Various features were extracted from the recorded videos through careful analysis, including the location of the hitting point, the subject’s posture, and the location of the hit ball. Overall, these cameras allowed for a detailed and accurate analysis of the participant’s performance during badminton strokes.

#### Survey data

**Fig. 5:**
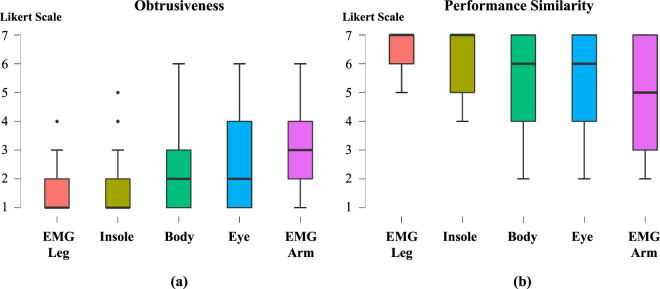
Survey Results.

In addition to collecting sensor data, we also gathered survey data on the physical attributes of the participants, periods of badminton training, and their experience with wearing sensors. We gathered fundamental metadata such as age, and gender, as well as biometrics such as weight, height, joint length, and dominant limbs from the participants. Furthermore, we obtained information on the duration of their professional training and their self-reported level of expertise to assess their skill levels. Moreover, to acquire information on the participants’ subjective experiences with wearing sensors, we collected data on the obtrusiveness of the five sensors and the performance similarity before and after wearing each sensor. The obtrusiveness refers to the extent to which a device or technology causes inconvenience, whereas the performance similarity is a metric that measures how closely a person’s regular badminton performance remains consistent, regardless of whether they are wearing a wearable sensor or not. By gathering data on the obtrusiveness of each of the five sensors and the performance similarity before and after wearing them, we were able to investigate how wearing the sensors affected the participants’ overall experience and data quality. The questionnaire was designed using a 7-point Likert scale, where higher values indicate greater levels of obtrusiveness and a higher degree of consistency in performance. The results showed that the Cognionics and Moticon sensors were associated with a low level of obtrusiveness, whereas the gForce and eye-tracking sensors were deemed to have relatively high obtrusiveness (see Fig. 5a). Despite the relatively high score for the latter two sensors, the majority of participants provided a score of 3 or less out of 7, indicating that the sensor devices did not cause significant discomfort. Regarding the performance similarity, the gForce sensor was reported to produce a low performance similarity, whereas the Cognionics and Moticon sensors demonstrated a high performance similarity (see Fig. 5b). In summary, the results suggest that wearing multiple sensors during the experiment did not significantly impact the participants’ wearable performance similarity or cause obtrusiveness. Moreover, the findings indicate that wearing the sensors did not compromise the quality of the collected data.

### Data annotation

**Fig. 6:**
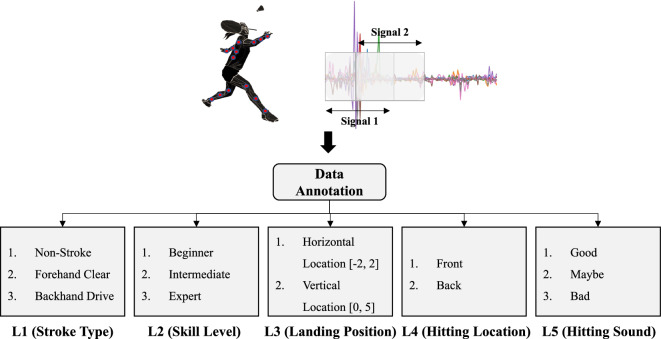
Annotation Levels.

In addition to the badminton data on stroke types, we proceeded with annotations of five hierarchical levels for the skill level, hitting sound, shuttlecock location, and hitting point, as shown in Fig. 6. In the case of the hitting point and hitting sound annotation among the five annotations, three independent labelers proceeded with annotation and verified the annotation data through inter-rater reliability. As mentioned in the Dataset Design section, badminton coaches are typically able to objectively evaluate badminton strokes based on factors such as stroke impact, ball trajectory, and contact point. To incorporate these perspectives into the development of a computer-aided evaluation system, we propose five levels of annotation.

#### Level 1 - Stroke type (Non-stroke, forehand clear, backhand drive)

We collected data on the types of strokes performed in badminton and annotated them accordingly. This involved identifying whether a stroke was a forehand clear, a backhand drive, or altogether not a stroke. We annotated the beginning and end of each stroke in real-time, using Unix time, while also recording the type of each stroke.

#### Level 2 - Skill level (beginner, intermediate, expert)

To annotate each participant’s skill level, we recruited three professional badminton coaches. Each coach had an average of 18 years of professional training and playing experience, with over 7 years of coaching experience (Age: Mean = 36.7, SD = 11.3; Years of Educational Experience: Mean = 18, SD = 2.6; Years of Coaching Experience: Mean = 8.2, SD = 1.3). These coaches were assigned the task of observing participants’ videos and rating their forehand clears and backhand drives on a scale ranging from 1 to 7. Here, a score of 1 represents a beginner, and 7 represents an expert. The dataset incorporated the individual scores from each of these three experts. Based on the average scores among three experts, participants were categorized as follows: those with an average score between 1 and less than 3 were classified as beginners, those with scores from 3 to less than 6 as intermediates, and those with scores of 6 or above as experts.

#### Level 3 - Horizontal and vertical landing position of the ball

We annotated the landing positions of the shuttlecock on the court in both horizontal and vertical dimensions. Prior to collecting stroke data, we asked participants to hit a shuttlecock toward the center of the court. By analyzing the landing position of the shuttlecock, we gathered information on the accuracy of the player’s stroke. As mentioned in the Dataset Design section, coaches often use the trajectory of the shuttlecock to evaluate a player’s training process and provide feedback. To capture the shuttlecock’s trajectory, we installed a camera to record the launch position, the participant, and the trajectory of the shuttlecock. Further, videos were recorded using Pupil Invisible glasses to determine the precise location of the shuttlecock landing. The horizontal position was annotated from −2 to 2, and the vertical position from 0 to 5, as depicted in Fig. 8, with the assigned numbers representing interval categories rather than meters. The left side of Fig. 8 illustrates the virtual baseline of the interval, while the right side shows physical lines that were utilized to create the virtual grid on the left. These data allowed us to analyze the accuracy of players hitting the shuttlecock at different locations on the court.

#### Level 4 - Hitting point (front, back, not contact)

To provide information on the stroke timing in relation to the player’s position and the shuttlecock, we annotated the point of contact, which indicates whether the shuttlecock was hit in front of or behind the player’s body. This factor is commonly utilized by expert coaches to assess a player’s badminton stroke posture and hitting accuracy. It is a generally taught principle that for optimal technique and power, the shuttlecock should be hit in front of the body, a point highlighted in the Dataset Design section. To record this, we installed a camera that captured a side view of the participant and filmed the participant throughout the data collection. Similar to other annotation levels, the location of the hit points in the dataset was annotated by three researchers using the recorded video footage. If the shuttlecock was hit in front of the body, it was marked as “Front”, and if it was hit from behind, it was marked as “Back”.

#### Level 5 - Stroke sound (good, maybe, bad)

We annotated the hitting sounds produced during strokes as “good”, “maybe”, or “bad” to provide insight into the quality of the stroke and the skill of the player. As mentioned in the interview responses of badminton coaches in the Dataset Design section, making a good shot depends on the strike impact of the racket on the shuttlecock, and the sound generated during a stroke is an important factor in evaluating a player’s skill. The sound of the stroke was collected using the sensor mounted on the Pupil Invisible glasses and annotated using the recorded eye video. To ensure the validity of annotations, three HCI researchers performed the annotations, and inter-rater reliability was later calculated to measure the consistency of the annotation values between the researchers. We classified the sound as “good” when it resembled the sound made when a professional player hits the shuttlecock squarely. A sound from a weak hit was classified as “maybe”, whereas a sound from a missed strike was classified as “bad”.

### Environment

**Fig. 7:**
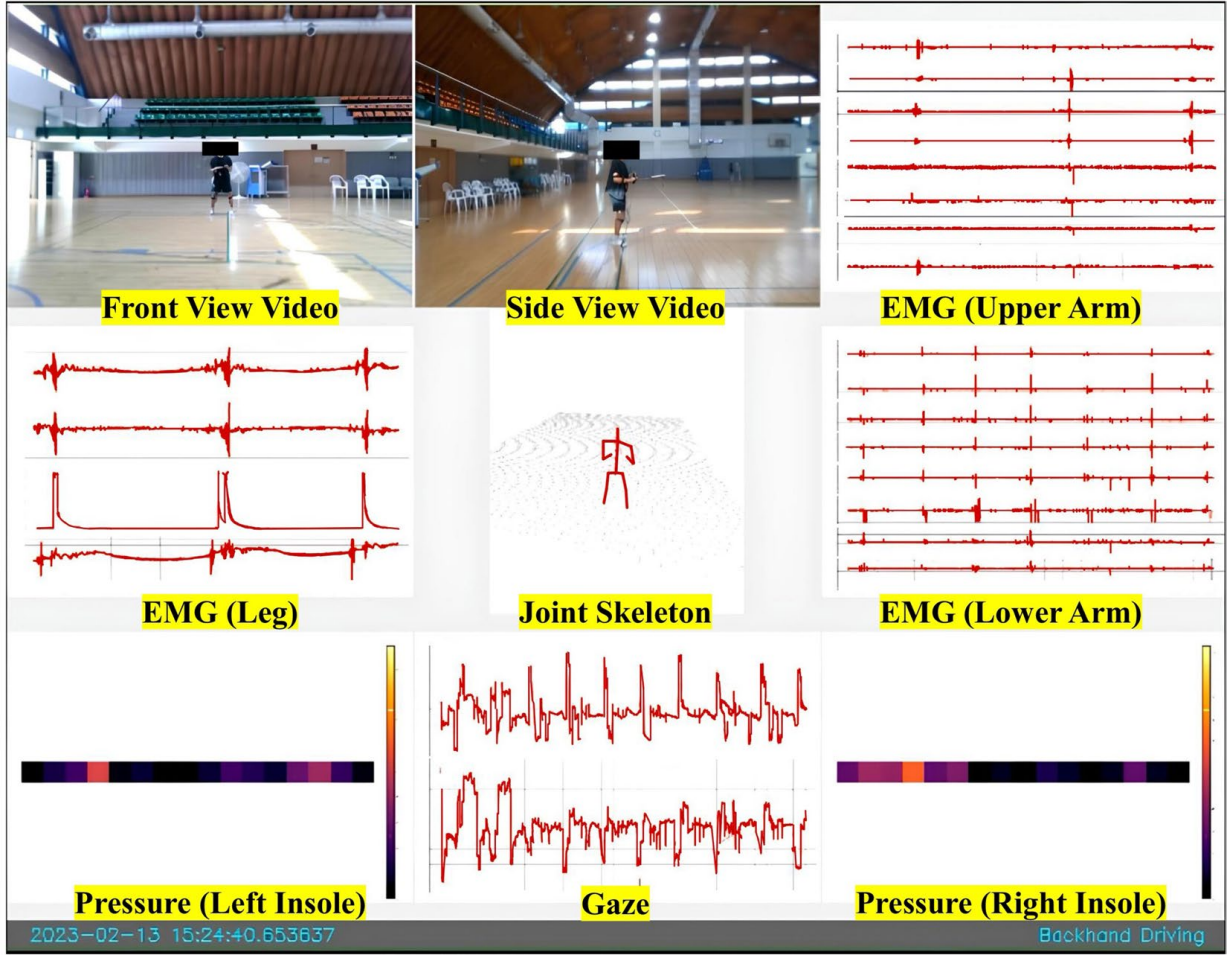
Real-time sensor-data visualization.

The data collection environment consisted of a shuttlecock launcher and three cameras that captured the front, side, and full views of the subjects (Fig. 4a,b). The shuttlecock launcher was positioned to ensure the ball always deployed in a constant trajectory according to the stroke type. The analyst was located at the edge of the badminton court with a computer for data collection and observed whether the collection of sensor data proceeded well. For efficient data observation, real-time visualization (demonstrated in Fig. 7) of the collected sensor data was applied to monitor the quality of the gathered data. Using this approach, the research team was able to identify any instances of sensor disconnection or poor attachment, as well as to detect drift in the IMU. In fact, some participants experienced such disruptions during the collection process, necessitating additional data collection.

**Fig. 8:**
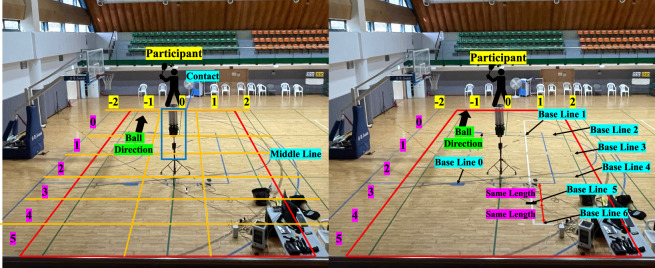
Instruction of Annotation Level 3: The left figure illustrates the virtual baseline of the interval, and the right figure shows physical lines that are utilized to create the virtual grid. In the case of Annotation Level 3, the ball landing location was annotated with a horizontal scale ranging from −2 to 2 and a vertical scale ranging from 0 to 5. The moment when the ball hit the shuttlecock launcher was annotated as “Contact’’.

### Data collection protocol

The data collection process is summarized in Table 4. First, the participants were briefed regarding the types of sensors they would be wearing, the number and types of strokes to be performed, and relevant instructions well in advance. The participants then completed a preliminary survey that gathered basic human and physical information along with their badminton experience. Subsequently, the participants wore five sensors and underwent a calibration process during which the sensors were connected to a Python framework. Once the calibration process was completed, the participants watched an instructional video on strokes and practiced hitting the shuttlecock at least five times while adhering to specific guidelines. The researcher provided guidance on the preparatory posture and instructed participants to hit the shuttlecock towards the center of the court. Both the calibration process and the practice stroke process have been stored in HDF5 format. Once the participants had mastered the preparatory posture, more than 150 data points were collected for each type of stroke in a randomized order that varied between participants. During data collection, the researcher continuously monitored the sensor collection visualization and conducted additional calibration in the event of missing sensor data or body-tracking drift. The researcher utilized a remote control to deploy a ball from the shuttlecock launcher, and based on this, annotated the start time, end time, and stroke type in real time. Upon completion of data collection, the participants were asked to complete a post-study survey to assess their level of comfort with the sensors, the extent to which their experience mirrored that of regular badminton play, and their willingness to have their data disclosed. The data collection process for each participant took approximately two-and-a-half hours to complete.

**Table 4:** Data Acquisition Procedure.

Step	Duration	Descriptions
1. Description and pre-survey	15 min	After a brief introduction to the sensor to be worn and the data collection process, a questionnaire on personal information was completed.
2. Wearing and calibrating sensors	20 min	Wearing five sensors and calibrating each sensor
3. Watching a video about the stroke	5 min	Watching a video about forehand clear or backhand drive.
4. Practice Strokes	3 min	Five or more warm-up strokes after receiving instruction on the ready position
5. Data Collection (150 strokes)	25 min	Collecting data for 150 rounds
6. Repeat steps 3 to 5 for other strokes	35 min	Collecting data for another 150 rounds
7. Post-survey	10 min	A post-survey on the experience of wearing the sensors was conducted.

## Data Records

The MultiSenseBadminton dataset^75^ is available on figshare. The collected dataset was a multimodal dataset that involved 25 participants of varying skill levels and had a total duration of 1,403 minutes (*Mean* = 56 minutes, *SD* = 7.48 minutes). In addition to physiological data such as EMG and gaze, the dataset contains behavioral data such as foot pressure and joint movement, as well as video data for sensor visualization and annotation data. The dataset also includes data summary files and survey data, in addition to sensor data.

**Fig. 9:**
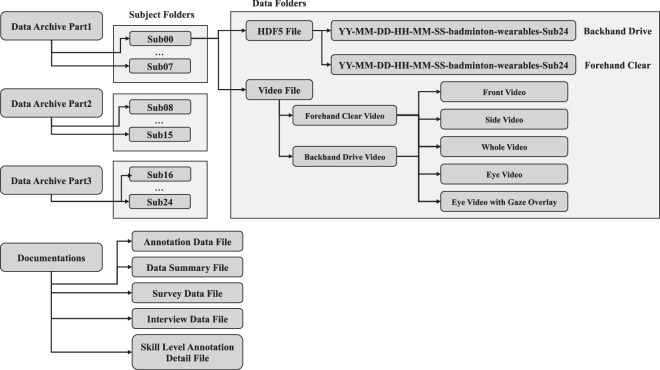
Hierarchical folder structure of the dataset.

All the data files are accessible on a hierarchical database, which facilitates navigation and retrieval of specific data. The organization of the dataset is depicted in Fig. 9, which provides a visual representation of the hierarchical structure of the dataset. The organization of the dataset follows a tree-like structure, with the top-level folder being an archive folder. The archive folder contains four types of files, a data-summary file, an interview file, an annotation data file, and a survey-data file, along with subject folders.

Each subject in the project has sensor-data HDF5 files labeled with the date of data collection and the participant ID. The HDF5 files can be easily accessed using HDF5 viewer software, and the Python code for reading these files is available on the project’s GitHub repository. The HDF5 file contains sensor stream data and Unix time.

The annotation file records the Unix time, stroke number, and annotation value in five levels, providing detailed information about various aspects of badminton strokes. The survey data and data-summary files contain information on all participants, such as demographic information and questionnaire responses, and can be used to understand the participants’ characteristics and study findings.

### HDF5 file details

The HDF5 file contains the following information: EMG values of leg, forearm, and arm start and stop time of strokes and calibration, eye gaze, foot pressure data, and motion capture data. Each sensor data structure is composed of “data”, “time s”, and “time str”. The “data” represents sensor data for each channel, “time s” represents Unix time for each data, and “time str” represents the global time for each data. The following subsections introduce the structure and composition of the dataset.

#### cgx-aim-leg-emg

This dataset encompasses EMG values for the dominant leg, expressed in millivolts (mV), across four distinct channels. Each channel serves to characterize specific muscle information, with the unit being mV: channel 1 is designated for the rectus femoris; channel 2 corresponds to the vastus medialis; channel 3 is allocated for the vastus lateralis; and channel 4 pertains to the biceps femoris.

#### experiment-calibration

The calibration dataset, with its two channels, relates to EMG data for the arm, forearm, and leg. The first channel marks the beginning and end of calibration. The second channel identifies the calibration pose used, either for gforce, involving three specific arm motions, or leg EMG, with two leg motions. The calibration pose for forearms includes lower arm inward pose and lower arm outward. And the calibration pose for the arm includes the upper arm inward pose. And the calibration poses for the leg include the leg forced pose and squat pose. The primary aim of this calibration is to obtain the maximum EMG value, aiding in the proper normalization and calibration of subsequent EMG measurements.

#### eye-gaze

This dataset contains two types of data: gaze data and worn data. The gaze data is designed to capture eye-tracking data through the use of pupil-invisible glasses and is artfully constructed into two channels. The first channel precisely maps the X-coordinate of gaze positions, spanning from 0 to 1088. This span aligns with the horizontal resolution of the video, facilitating accurate monitoring of gaze movements across the horizontal axis. The second channel records the Y-coordinate of gaze positions, extending from 0 to 1080, reflecting the vertical resolution of the video. This enables a thorough examination of vertical gaze movements. In addition to these channels, the dataset includes a “worn” column. This column is designed to indicate whether the glasses were worn during the data capture, with a value of 1 denoting the glasses were on and a value of 0 indicating their absence. This binary value provides a clear indication of the presence or absence of the glasses, thereby informing the status of data prediction during the capture process.

#### gforce-lowerarm-emg

This dataset furnishes EMG values for the lower arm, represented in normalized units ranging between 0 and 250 across eight channels.

#### gforce-upperarm-emg

Analogous to the gforce lower arm dataset, this collection entails EMG values for the upper arm, comprising eight channels, with data normalized between 0 and 250.

#### moticon-insole

The Moticon insole data contains five types of data. The first type of data is the Center of Pressure (COP). It is represented by four channels denoting the x and y coordinates of COP for both feet. The first channel denotes the COP x coordinates of the left foot, the second channel denotes the COP y coordinates of the left foot, the third channel denotes the COP x coordinates of the right foot, and the fourth channel denotes the COP y coordinates of the right foot. The second type of data is the acceleration. It captures the linear acceleration of the foot across the x, y, and z directions, expressed in g (gravity). The first channel is the acceleration of x coordinates, the second is the acceleration of y coordinates, and the third is the acceleration of z coordinates. The third type of data is the angular velocity. It captures the angular velocity of the foot across the x, y, and z directions, measured in degree/s. The first channel is the angular velocity of x coordinates, the second is the angular velocity of y coordinates, and the third is the angular velocity of z coordinates. The fourth type of data is the pressure. It captures the pressure maps around the foot in 16 channels, measured in N/cmÂ². The fifth type of data is the total force. It captures the total force of each foot in 1 channel, measured in Newton (N).

#### pns-joint

This dataset contains four types of data: information relative to joint global positions, joint local positions, quaternions, and Euler angles, encompassing the following parameters for 21 joints. And the order of the joints within the dataset is detailed as follows: hip, right up leg, right leg, right foot, left up leg, left leg, left foot, spine, spine 1, spine 2, neck, neck 1, head, right shoulder, right arm, right forearm, right hand, left shoulder, left arm, left forearm, left hand.

The first type of data pertains to the local position of each joint, measured in centimeters (cm). This data is structured across a total of 63 channels, comprising the x, y, and z coordinates for each joint. The second type of data pertains to the global position of each joint, measured in centimeters (cm). This data is structured across a total of 63 channels, comprising the x, y, and z coordinates for each joint. The third type of data pertains to the Euler angle of each joint, measured in degrees. This data is structured across a total of 63 channels, comprising the x, y, and z coordinates for each joint. The fourth type of data pertains to the quaternion of each joint, measured in degrees. This data is structured across a total of 84 channels, comprising the w, x, y, and z values for each joint.

### Video and document file details

In our dataset, we have included five distinct types of video recordings of the participants, capturing various perspectives: Front Video, Side Video, Whole Video, Eye Video, and Eye Video with Gaze Overlay. To protect the privacy of the participants, these videos have been made anonymous, with some editing done to make sure that the participants cannot be identified. It should be noted that the Eye Video and Eye Video with Gaze Overlay contain appearances by the research team that are not anonymized; however, the researchers have given their consent for the release of this identifiable data. We also included survey data, annotation data, data summary files, and interview data in document files, in addition to sensor and video data from participants. The detailed descriptions of each file are as follows:

#### Data summary file.xlsx

This file summarizes the collection status of sensor and video data for each participant. Each column includes the participant’s name, file name, stroke type, whether calibration is included, the inclusion of data from each sensor, and the inclusion of each type of video. A “circle” indicates that the data is included, an “X” signifies that the data is missing, and a “triangle” represents that some of the data is partially missing.

#### Annotation data file.xlsx

This file marks each stroke at the annotation level for each participant. Each column contains detailed information on the subject number, annotation start time, annotation stop time, stroke number, and annotation level. Especially for annotation levels 4 and 5, the file also includes evaluations made by three raters for each level.

#### Skill level annotation detail file.xlsx

The file contains data summarizing the skill level assessments for each clear and drive stroke by three expert badminton coaches. Each column includes the participant number, the skill level for each stroke, the reason for the skill level assessment, and annotation data indicating whether the participant was classified as a beginner, intermediate, or expert in our dataset.

#### Survey data file.xlsx

The file includes survey information on participants’ basic personal information, physical attributes, and experience with data collection. Each column contains data on the participant number, age, gender, weight, dominant hand, dominant leg, lengths of 13 different joints, professional training experience, self-reported expertise level, experience with sensor attachment, and consent for data disclosure.

#### Interview data

The file summarizes the questions and answers from a discussion with three experts prior to collecting badminton data. It includes topics such as their usual training processes, important aspects of badminton coaching, and methods of providing feedback.

## Technical Validation

### Examining missing data

The availability of data for each participant is summarized in a data summary file. The file indicates whether calibration data are present in each participant’s data folder, and whether sensor data from five sensors and video data are available. An “O” indicates data are available whereas an “X” indicates absence of data. Blank spaces represent data that were not originally collected.

Regarding wearable sensors, all sensor data are accessible, except for eye-tracking data from participants labeled Sub05, Sub06, and Sub08. For these participants, the eye-tracking camera was either broken, the gaze data were missing, or the gaze data were not collected; therefore, these data were excluded from the available data. Also, some videos were not recorded owing to program interruption during data collection. In preparation for this, multiple cameras were used to record concurrently to minimize missing annotation data. Nonetheless, when eye video data were not recorded, sound data were likewise not recorded, hence some data were missing in this regard. For Annotation Level 3, only 61 out of 7763 annotations were missing, accounting for 0.78% of total annotations. For Annotation Level 4, 19 annotations were missing, representing 0.24% of the total. Finally, in the case of Annotation Level 5, there were 857 missing annotations, accounting for 11.04% of the total annotations.

During the data collection process, some video data was missing. For the Side Video, data was missing for Sub00, Sub03, and Sub04. In the case of the Whole Video, data for Sub00, Sub03, Sub04, Sub05, and Sub15 was missing. For the Eye Video, there were missing data for Sub05, Sub10, Sub12, and Sub16. And for the Eye Video with Gaze Overlay, data was missing for Sub02, Sub05, Sub06, Sub07, Sub08, and Sub13. Due to these missing pieces, approximately 10% of the video data, which amounts to 25 videos, could not be collected, resulting in a total of 225 videos.

### Evaluating inter-rater reliability in skill level annotation

In video-based annotation, we analyzed inter-rater reliability across three skill levels: beginner, intermediate, and expert. Inter-rater reliability refers to the extent of agreement or consistency among different raters or annotators when assessing or scoring the same dataset. We calculated the inter-rater reliability between two coaches and among three coaches using Cohen’s kappa and Fleiss’ kappa values, respectively. Cohen’s kappa is a statistic used to measure the agreement between two raters, going beyond mere chance agreement. Fleiss’ kappa, on the other hand, is an extension of Cohen’s kappa for measuring the agreement between three or more raters.

Table 5 displays the inter-rater reliability among coaches, as determined by Cohen’s Kappa and Fleiss’s Kappa values, and the number of participants classified as beginners, intermediates, and experts through this annotation. Overall, the inter-rater reliability among the coaches showed a moderate level of agreement, with values above 0.64.

**Table 5:** Skill level annotation; # means the number of subjects.

Stroke	# Beginner	# Intermediate	# Expert	IRR (R1&R2)	IRR (R1&R3)	IRR (R2&R3)	IRR (ALL)
Clear	11	8	6	0.70	0.63	0.59	0.64
Drive	11	9	5	0.82	0.82	0.64	0.75

### Annotation distributions and inter-rater reliability for annotation levels 4 and 5

**Fig. 10:**
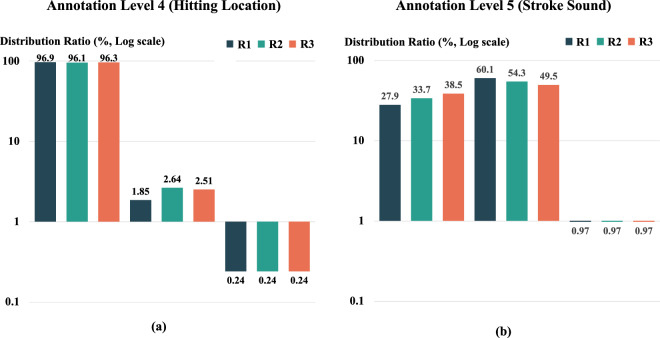
Distributions and frequencies of Annotation Levels 4 and 5 from three raters (R1, R2, R3): Not Contact indicates the shuttlecock and the racket do not make proper contact. The graph is designed with a logarithmic scale on the y-axis to account for data imbalance.

The distribution and frequency of annotations are shown in Fig. 10a,b, where the term “Not Contact” indicates that the participant did not make contact with the ball. The total number of stroke instances is 7,763 and we expressed each label as a percentile ratio.

To estimate inter-rater reliability of Annotation Level 4 and 5, Krippendorff’s alpha was calculated. Krippendorff’s alpha is widely recognized as the most versatile reliability measure, particularly when not all strokes are assessed by every rater^88,89^. We also calculated the percentage of agreement for the three levels of annotation by determining the number of instances in which all three annotators assigned the same annotation, divided by the total number of annotations.

**Fig. 11:**
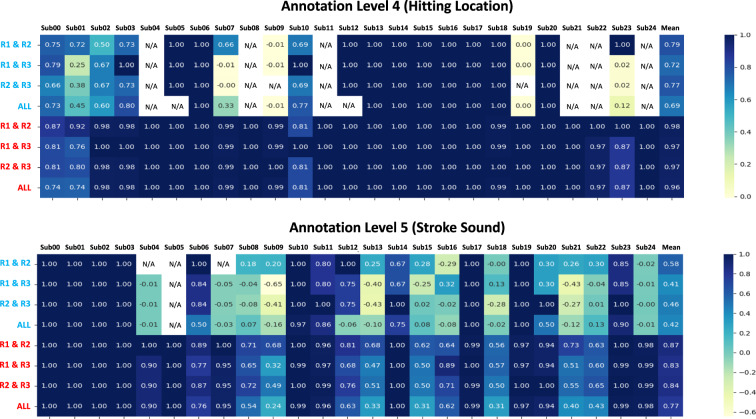
Heat maps of inter-rater reliability measured by Krippendorff’s alpha and percentage of agreement for each subject: The first four rows (blue text) indicate Krippendorff’s alpha value, while the last four rows (red text) indicate the percentage of agreement. The columns show the respective metrics for each participant, and R1, R2, and R3 represent the raters.

Figure 11 displays a heat map of Krippendorff’s alpha coefficients and percentage of agreement calculated for three annotation levels measured on an ordinal scale. Annotation Levels 4 and 5 had high agreement percent (0.96 and 0.77, respectively), indicating a good agreement score. However, when considering Krippendorff’s alpha value, Annotation Level 5 received a low score of 0.42. This can be attributed to the subjective nature of the task, where raters assessed the quality of a sound as Good, Maybe, or Bad. The final annotation data files for levels 4 and 5 contain both the consolidated annotation values that received agreement from multiple raters and the raw annotation values provided by each individual rater.

### Preliminary learning pipelines

In this section, we provide an initial pipeline for utilizing our multi-modal data, aiding easier use of our dataset for non-expert developers. Classification results for each annotation are demonstrated with an state-of-the-art neural network model. This structure can be tailored to fit individual researchers’ unique hypotheses and objectives. Much of the pipeline’s foundation is drawn from the ActionSense dataset^76^, from which we adapted preprocessing and analysis methodologies. The experimental methods and the results for dataset validity are elaborated are provided in the following sections.

#### Data preprocessing and feature extraction

**Fig. 12:**
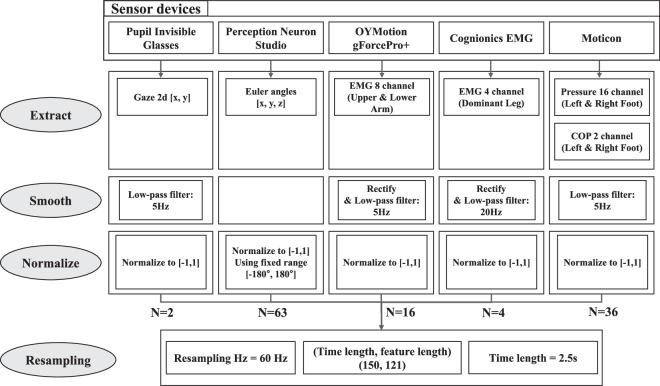
Data Preprocessing.

We derived six types of features from five distinct types of wearable sensors. These features encompassed gaze 2D data obtained from Pupil Invisible Glasses, joint Euler angles extracted from Perception Neuron Studio, EMG data collected from gForcePro + and Cognionics EMG sensors, as well as pressure and center of pressure data acquired from Moticon sensors. To synchronize these features, each sensor was integrated into the main computer’s data collection framework. This involved fetching the Unix time and data from each sensor server and transmitting these simultaneously. Based on this approach, the Unix time and sensor data were collected simultaneously for five different sensors. We performed preprocessing for each sensor, and the preprocessing process is summarized in Fig. 12. To facilitate further research in this area, the code used in this study for data preprocessing and classification was published on GitHub as an open-source project.

In the preprocessing stage, we extracted features from five sensors: 2 channels of 2D gaze data, 63 channels from 21 joint Euler angles, 16 channels for upper and lower arm EMG, 4 channels for leg EMG, and 36 channels from insole sensors, which include both 32 pressure channels and 4 COP channels. To reduce noise and artifacts in the data, we applied a low-pass filter with cut-off frequencies of 5 Hz for the arm EMG, 2D gaze and insole pressure, and 20 Hz for the leg EMG. The EMG value was rectified by taking its absolute value before applying a low-pass filter. Altogether, this process generates a total of 121 data channels, with each channel normalized within the range of [−1, 1]. Subsequently, we resampled all channels to a uniform time vector at a 60 Hz sampling rate, employing linear interpolation throughout to ensure consistent temporal alignment across the data. By segmenting each stroke example with a 2.5-second interval, we extracted a total of 7761 stroke instances from 18 participants. This total includes 2607 instances of backhand drive, 2613 instances of forehand clear, and 2541 instances of non-strokes, where non-strokes refer to instances where no stroke was being performed. Utilizing our stroke dataset, we proceeded to classify the five types of annotations we collected; types of strokes, skill level, horizontal location, vertical location, hitting point, and sound.

#### Network architecture

For our training process, we constructed models using deep learning architectures commonly employed for time-series data: ConvLSTM, Long Short-Term Memory (LSTM), and Transformer. These models were developed to effectively capture and analyze the temporal patterns inherent in our dataset. Our pipeline, implemented in Python 3.9 using Pytorch, utilized these architectures to accurately extract key features from sensor data for sequential information classification. We consistently applied these architectures across the classification of annotation data.

To evaluate the model’s performance, we used Accuracy, Balanced Accuracy, and F1 score as metrics, with the Adam optimizer and categorical cross-entropy as the loss function. The model’s performance was assessed using hyperparameters: learning rates of 0.0005 and 0.0001, and epochs of 200. We applied early stopping with a patience of 10. The dataset division into train + and test sets involved two validation methods: 10-fold cross-validation, where the dataset is split into ten equal parts, each part used once as a test set while the others serve as training data, ensuring all data is used in both roles; and leave-three-out (LTO) cross-validation, which involves selecting one subject from each skill level - beginner, intermediate, and expert - and using their data as the test set, while data from other subjects form the training set. Detailed reference numbers for subjects used in the LTO cross-validation, which demonstrate the unique combinations of participants from different skill levels–beginner, intermediate, and expert–across ten iterations, are listed in Table 6. This approach tests the model’s ability to generalize to new subjects across different skill levels. Additionally, for comparison, we developed a baseline model that predicts the predominant class, serving as a benchmark to assess our models’ performance and effectiveness against this basic approach.

**Table 6:** Leave-Three-Out reference subject number; This table provides the reference subject numbers for the LTO cross-validation process, facilitating a reproducible benchmark.

Iteration Number	Skill Level			Iteration Number	Skill Level		
	Beginner	Intermediate	Expert		Beginner	Intermediate	Expert
1	Sub00	Sub09	Sub11	6	Sub04	Sub09	Sub11
2	Sub01	Sub15	Sub14	7	Sub07	Sub15	Sub14
3	Sub02	Sub18	Sub19	8	Sub17	Sub18	Sub19
4	Sub03	Sub21	Sub20	9	Sub23	Sub21	Sub20
5	Sub04	Sub22	Sub24	10	Sub00	Sub22	Sub24

#### Stroke type and skill level classification results

In the case of stroke type classification, the study involved categorizing three types of strokes (forehand clear, backhand drive, and non-stroke). Table 7 displayed the mean and standard deviation of accuracy, balanced accuracy, and F1 score, obtained through LTO validation. Overall, deep learning models outperformed the baseline in all metrics, with ConvLSTM demonstrating particularly superior performance across all metrics compared to other models.

**Table 7:** Stroke Type Classification Results; models with the highest performance in each metric are highlighted in bold.

Annotation	Model	LTO Results		
		Acc_*avg*_ (SD)	BalAcc_*avg*_ (SD)	F1_*avg*_ (SD)
Stroke Type	Transformer	83.15 (7.53)	83.16 (7.52)	82.85 (7.82)
	ConvLSTM	**90.03 (6.92)**	**90.07 (6.91)**	**89.99 (6.96)**
	LSTM	82.85 (6.86)	82.85 (6.84)	82.83 (6.88)
	Baseline	33.89 (0.22)	33.33 (0.00)	17.16 (0.19)
Skill Level_*clear*_	Transformer	40.60 (10.27)	41.84 (10.72)	40.11 (12.21)
	ConvLSTM	45.69 (11.79)	45.87 (12.66)	44.50 (16.23)
	LSTM	**46.56 (8.65)**	**48.35 (11.73)**	**44.92 (10.28)**
	Baseline	34.53 (0.55)	33.33 (0.00)	17.73 (0.49)
Skill Level_*drive*_	Transformer	42.60 (11.36)	42.71 (11.43)	39.17 (9.45)
	ConvLSTM	46.39 (12.46)	46.33 (12.46)	42.02 (15.27)
	LSTM	**46.98 (12.92)**	**47.02 (12.86)**	**44.56 (13.16)**
	Baseline	33.79 (0.23)	33.33 (0.00)	17.07 (0.20)

In the case of skill level classification, the study involved categorizing three types of strokes (Beginner, Intermediate, and Expert), shown in Table 7. Overall, deep learning models outperformed the baseline in all metrics, with LSTM demonstrating particularly superior performance across all metrics compared to other models.

#### Annotation classification results for clear

In the classification of annotation data related to clear strokes, Table 8 presents an analysis results using different models, evaluated through both LTO and 10-fold cross-validation methods. In the horizontal landing location classification, the Transformer model stood out in the LTO results, with its efficacy closely matched in the 10-fold setting. In the vertical landing position classification, the baseline model unexpectedly outperformed others in the LTO approach. This could be attributed to the vertical landing position being relatively consistent across players of different skill levels, leading to higher baseline accuracy as the data for this category is less variable. Additionally, in the hitting point classification, a similar trend was observed where the baseline accuracy was significantly high, likely due to the predominant distribution of data being classified as “Front”, skewing the results. However, in the 10-fold cross-validation for vertical landing position, the LSTM model demonstrated superior performance. The hitting point classification showed strong performances from both the ConvLSTM and LSTM models, with the latter slightly edging out in the 10-fold cross-validation. Lastly, in the sound classification, the ConvLSTM model exhibited the best performance in all metrics in the LTO approach, while in the 10-fold cross-validation, the LSTM model led in both accuracy and F1 score.

**Table 8:** Clear Classification Results; models with the highest performance in each metric are highlighted in bold.

Annotation	Model	LTO Results			10-Fold Results		
		ACC_*avg*_ (SD)	BalACC_*avg*_ (SD)	F1_*avg*_ (SD)	ACC_*avg*_ (SD)	BalACC_*avg*_ (SD)	F1_*avg*_ (SD)
Horizontal_*clear*_	Transformer	**79.47 (4.83)**	**24.37 (3.90)**	**70.49 (6.59)**	**79.42 (3.28)**	27.73 (3.93)	**73.96 (4.26)**
	ConvLSTM	70.94 (10.23)	25.50 (5.66)	69.05 (6.96)	61.35 (7.55)	**32.77 (4.10)**	63.92 (6.76)
	LSTM	77.62 (4.43)	24.68 (3.96)	70.91 (5.16)	76.93 (3.29)	28.90 (2.88)	73.86 (3.70)
	Baseline	79.45 (4.84)	24.33 (3.94)	70.43 (6.63)	79.06 (2.78)	23.50 (2.42)	69.84 (3.82)
Vertical_*clear*_	Transformer	37.27 (10.70)	15.39 (3.68)	33.19 (13.70)	**75.51 (3.11)**	49.28 (4.62)	73.72 (3.16)
	ConvLSTM	31.64 (14.54)	**18.30 (8.25)**	30.36 (16.36)	68.10 (5.31)	**52.79 (2.95)**	68.66 (3.89)
	LSTM	36.99 (8.44)	15.52 (2.62)	33.60 (11.38)	75.34 (2.65)	51.19 (6.46)	**73.94 (3.09)**
	Baseline	**55.99 (7.24)**	15.90 (2.37)	**40.45 (8.40)**	39.08 (1.99)	14.52 (0.75)	21.99 (1.92)
Hit Point_*clear*_	Transformer	95.38 (6.66)	48.32 (19.95)	93.25 (9.73)	95.11 (1.77)	53.28 (10.08)	94.56 (2.07)
	ConvLSTM	81.16 (9.99)	42.75 (15.70)	85.47 (7.63)	78.87 (8.86)	**62.34 (10.98)**	84.29 (6.26)
	LSTM	95.29 (6.60)	48.25 (19.76)	93.21 (9.70)	**95.25 (1.20)**	49.84 (11.02)	**94.59 (1.75)**
	Baseline	**95.40 (6.67)**	**48.33 (19.95)**	**93.26 (9.73)**	94.53 (1.22)	35.00 (5.27)	91.88 (1.79)
Sound_*clear*_	Transformer	56.25 (8.01)	39.63 (7.55)	53.38 (8.98)	86.65 (1.67)	62.40 (10.36)	86.35 (1.85)
	ConvLSTM	**64.23 (15.88)**	**47.82 (19.09)**	**60.89 (17.58)**	85.61 (4.94)	**65.89 (12.65)**	85.45 (4.93)
	LSTM	59.87 (8.05)	42.36 (12.23)	58.00 (8.68)	**88.38 (3.04)**	59.23 (2.04)	**88.08 (3.01)**
	Baseline	54.48 (3.03)	35.00 (5.27)	38.47 (3.54)	57.47 (2.74)	36.67 (7.03)	41.98 (3.28)

#### Annotation classification results for drive

Table 9 presents results across different models, evaluated using both LTO and 10-fold cross-validation. In the horizontal landing position classification, the performance varied between the LTO and 10-fold settings. In the 10-fold cross-validation, the Transformer model achieved the highest accuracy, while the ConvLSTM model had the highest balanced accuracy, and the LSTM model led in F1 score. In contrast, in the LTO results, the baseline model achieved the highest accuracy, ConvLSTM led in balanced accuracy, and the Transformer model scored the highest in F1 score. For the vertical landing position classification, the baseline model’s superior performance in the LTO approach can be attributed to the relatively consistent vertical landing positions across different players, resulting in less variable data and therefore higher baseline accuracy. In contrast, in the 10-fold cross-validation, the LSTM model showed the highest balanced accuracy and F1 score for vertical landing position. In the hitting point classification, the majority of the data was distributed in the “Front” category. This predominance led to no significant performance difference between the baseline and the top-performing models across two validation settings. Lastly, in the sound classification, varied performances were observed among the models. The Transformer model excelled in the 10-fold cross-validation, while the ConvLSTM and LSTM models displayed strong results in the LTO settings, particularly with LSTM leading in LTO for F1 score and accuracy.

**Table 9:** Drive Classification Results; models with the highest performance in each metric are highlighted in bold.

Annotation	Model	LTO Results			10-Fold Results		
		ACC_*avg*_ (SD)	BalACC_*avg*_ (SD)	F1_*avg*_ (SD)	ACC_*avg*_ (SD)	BalACC_*avg*_ (SD)	F1_*avg*_ (SD)
Horizontal_*drive*_	Transformer	68.88 (8.26)	22.46 (5.56)	**57.71 (10.73)**	**73.99 (3.04)**	37.70 (11.59)	70.93 (3.80)
	ConvLSTM	39.74 (13.02)	**24.81 (7.06)**	39.57 (12.92)	56.21 (6.49)	**41.96 (8.75)**	58.36 (6.12)
	LSTM	64.61 (6.13)	23.07 (8.38)	57.64 (9.62)	73.89 (2.16)	37.11 (6.03)	**71.51 (2.95)**
	Baseline	**69.82 (8.32)**	22.33 (5.34)	57.67 (10.98)	70.19 (3.18)	23.00 (2.58)	57.93 (4.14)
Vertical_*drive*_	Transformer	38.85 (11.60)	20.75 (5.91)	42.48 (11.32)	75.32 (2.19)	44.28 (2.58)	73.73 (1.95)
	ConvLSTM	47.40 (19.98)	21.32 (4.47)	**44.63 (19.47)**	75.21 (2.08)	**48.70 (5.48)**	73.87 (2.01)
	LSTM	42.95 (18.07)	**23.02 (5.37)**	44.02 (18.60)	**75.82 (3.33)**	48.35 (7.28)	**74.93 (3.49)**
	Baseline	**50.01 (15.09)**	16.05 (1.82)	34.57 (16.63)	42.19 (2.40)	14.52 (0.75)	25.08 (2.45)
Hit Point_*drive*_	Transformer	**99.15 (0.65)**	**50.00 (19.25)**	**98.73 (0.98)**	**99.06 (0.64)**	53.33 (17.21)	**98.59 (0.96)**
	ConvLSTM	89.36 (9.27)	44.17 (13.28)	93.40 (5.05)	89.31 (6.54)	**54.67 (17.35)**	93.43 (3.58)
	LSTM	**99.15 (0.65)**	**50.00 (19.25)**	**98.73 (0.98)**	**99.06 (0.60)**	46.67 (7.03)	**98.59 (0.89)**
	Baseline	**99.15 (0.65)**	**50.00 (19.25)**	**98.73 (0.98)**	**99.06 (0.54)**	51.67 (18.34)	**98.59 (0.81)**
Sound_*drive*_	Transformer	60.01 (11.69)	39.17 (7.67)	57.37 (11.92)	**90.08 (1.07)**	**59.58 (0.98)**	**89.63 (1.21)**
	ConvLSTM	62.40 (14.67)	**43.71 (9.47)**	59.79 (16.39)	83.45 (6.90)	58.37 (4.68)	83.57 (6.67)
	LSTM	**64.39 (10.74)**	41.56 (9.43)	**60.24 (12.89)**	89.79 (1.68)	59.48 (1.19)	89.37 (1.68)
	Baseline	59.27 (5.73)	35.00 (5.27)	44.26 (6.86)	65.74 (2.61)	35.00 (5.27)	52.17 (3.36)

The provided pipeline represents a preliminary implementation of a deep-learning application to evaluate the suitability of the MultiSenseBadminton sensor and annotation dataset^75^. An important consideration in our study is the imbalance in data distribution across participants, particularly evident in the annotations for horizontal landing position, vertical landing position, and hitting point. This imbalance inherently leads to higher baseline accuracy in certain cases, as the majority class tends to dominate the dataset. This phenomenon is especially notable in the vertical position and hitting point classifications, where the baseline accuracy occasionally surpasses that of the deep learning models. Such an outcome underscores the challenges posed by imbalanced data in machine learning, especially in the context of sports analytics where participant variability can significantly impact model performance. It highlights the need for careful consideration of data distribution and participant variability when interpreting model accuracy and effectiveness in classifying stroke-related annotations.

## Usage Notes

### Sensor-data visualization

**Fig. 13:**
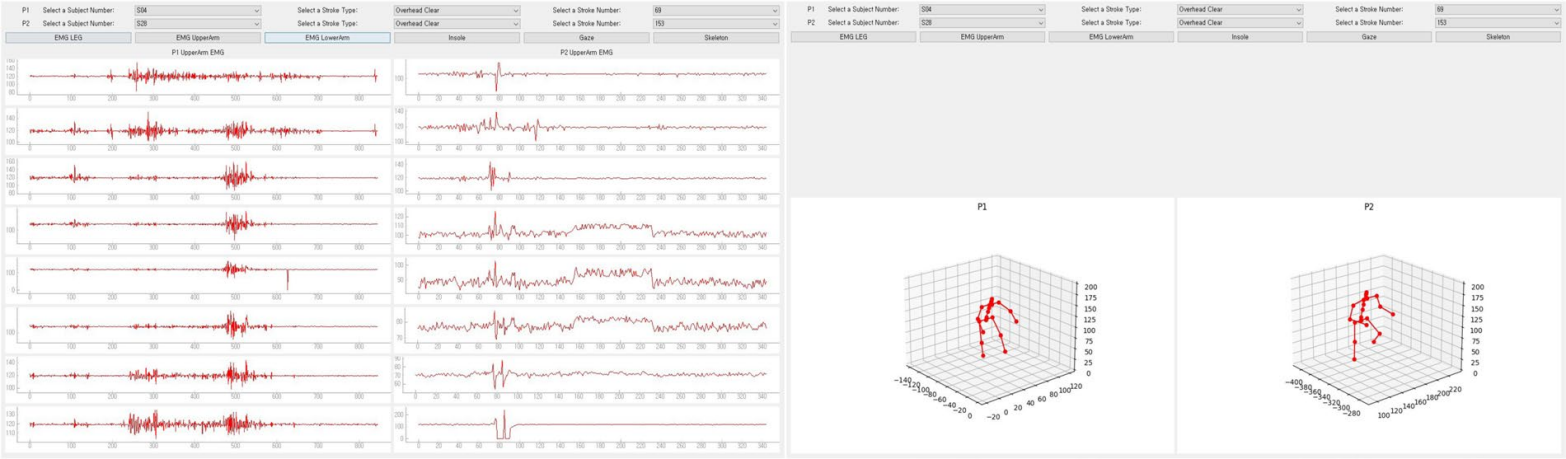
Data Visualizer.

We developed a data visualization tool to visualize sensor data and enable visual comparison of strokes, as shown in Fig. 13. The tool code is available on the project’s GitHub page, providing the capability to select various parameters for comparison, such as participant number, stroke type, stroke number, and sensor. These features allow for a comprehensive analysis of stroke patterns by facilitating the comparison of sensor data across different participants and strokes.

### Data limitations

The MultiSenseBadminton dataset^75^ has certain limitations that need to be addressed. First, some of the wearable sensors used in this study were susceptible to noise, drift, and connectivity issues during data collection. For instance, the body tracking sensor caused position drift, which is a common problem with IMU-based motion tracking sensors^90,91^. Although we mitigated this issue by calibrating the motion tracking sensors for each session and recalibrating them upon detection of discrepancies between participants’ actual movements and the corresponding joint data, the inherent nature of the IMU sensor inevitably introduces some drift-related error. Further, the Cognionics AIM sensor generated spike values during the stroke, and we therefore wrote preprocessing code to process the spike values. The preprocessing code has been uploaded to the GitHub page. There was also an interruption in the streaming of data from the gForce EMG armband during the data collection process. To address this issue, the badminton stroke was stopped and the stream was reconnected to resume data collection. This ensured that the collected data corresponded to actual badminton strokes and were free from interruptions or inaccuracies.

The second limitation of the MultiSenseBadminton dataset^75^ is that the data were collected in a constrained environment, designed to mimic a typical badminton coaching scenario. In this setup, participants responded to shuttlecocks launched by a machine, similar to how a coach might throw shuttlecocks to a trainee. This method ensured that shuttlecocks were delivered in a uniform trajectory, allowing us to gather consistent sensor data across various aspects such as movement, muscle activity, and center of pressure shifts among players of different skill levels. While this approach is useful for controlled training exercises, future studies should aim to collect data in real-world match environments. In this real-match scenario, it’s essential to use wearable sensors that do not restrict the participants’ movement. Therefore, we recommend utilizing non-intrusive sensors, such as insole-based foot pressure sensors and cameras, to ensure free and natural player movement. Additionally, for a more effective and realistic data collection, it is advisable to recruit participants with intermediate or higher skill levels who are capable of engaging in actual gameplay. This method of data collection in real-match settings will provide insights into player strategies and movements, enhancing the understanding of competitive badminton dynamics.

Third, the location of the wearable sensor presented limitations that prevented the collection of whole-body data on badminton strokes. The sensor was attached to a specific part of the body, which restricted data collection to that particular area. As a result, we were unable to obtain a comprehensive understanding of the mechanics of badminton strokes across the entire body. For instance, the use of wearable sensors in this study was limited by the number of available sensors, and as a result, EMG sensors were only attached to the dominant arm and foot. Moreover, the discomfort associated with wearing multiple sensors can affect the accuracy of badminton stroke data. This is a significant limitation of wearable sensors that needs to be addressed. In fact, some participants in the study reported various discomforts when wearing eye-tracking glasses and EMG armbands. They also reported slightly different experiences when sporting sensors during badminton motion than when not wearing any. Therefore, after collecting such data, it is essential to conduct additional research to reduce the number of sensors by analyzing the correlations between them.

Fourth, the use of video-based annotation limits the accuracy of annotation data. We used three cameras to record participants from different angles during data collection. As it was difficult to record aspects such as the hitting sound, hitting point, and landing position during the data collection process, the annotations for Levels 3, 4, and 5 were conducted after the data collection by three annotators. The inter-rater reliability was assessed to ensure the accuracy of the annotations. The inter-rater reliability value for Level 3 was relatively low owing to the subjective nature of the hitting sound. Moreover, even in the case of Levels 4 and 5, some annotations had missing values, or the agreement between annotators was low. To overcome these limitations, future research should focus on developing a system that can automatically detect ball trajectories and hit points. This would significantly improve the accuracy and reliability of annotation data and provide researchers with more comprehensive insights into the mechanics of badminton strokes.

Finally, our dataset focused on only two strokes, the forehand clear and backhand drive. Since our dataset’s objective was to assess the quality of badminton strokes across all skill levels, from beginners to experts, we concentrated on these two fundamental strokes. However, we recognize that focusing exclusively on these two strokes is a limitation. Moving forward, it is crucial to target players at intermediate levels and above, who are proficient in a broader range of advanced badminton techniques. Our future aim is to build a dataset for evaluating the quality of strokes involving more complex techniques such as hairpin, net shots, smashes, and drop shots, thereby expanding the scope and utility of our research in badminton stroke quality assessment.

## Data Availability

Software is available on GitHub and can be accessed via the following link. https://github.com/dailyminiii/MultiSenseBadminton. This comprehensive software package includes examples for reading and parsing HDF5 files, performing data preprocessing by extracting and filtering, and displaying the results. Additionally, it offers functionality for training a deep-learning model using the preprocessed data, generating a T-SNE plot based on the preprocessed data, and creating a visualization video based on the raw data presented in Fig. 13.
